# Race, Depression, and Financial Distress in a Nationally Representative Sample of American Adults

**DOI:** 10.3390/brainsci9020029

**Published:** 2019-01-30

**Authors:** Shervin Assari

**Affiliations:** 1 Department of Family Medicine, Charles R. Drew University of Medicine and Sciences, Los Angeles, CA 90095, USA; assari@umich.edu; Tel.: +734-647-7944; 2 Department of Psychiatry, School of Medicine, University of Michigan, Ann Arbor, MI 48109-2029, USA; 3 Center for Research on Ethnicity, Culture and Health (CRECH), School of Public Health, University of Michigan, Ann Arbor, MI 48109-2029, USA; 4 Department of Psychology, University of California Los Angeles, Los Angeles, CA 90095, USA

**Keywords:** depression, mood sisorders, African Americans, Blacks, ethnic groups, ethnicity, race, financial distress, financial hardship, financial insecurity, class, Socioeconomic Status (SES)

## Abstract

**Background:** Although depression and financial distress are correlated, this association may differ for demographic groups, particularly based on race. **Aim:** Using a national sample of American adults, this study tested whether the association between Major Depressive Episode (MDE) and financial distress differs between African Americans and Whites. **Methods:** The National Survey of American Life (NSAL), 2003, enrolled 3570 African American and 891 Non-Hispanic White American adults. Demographic data (age and gender), socioeconomic position (SEP; i.e., education, employment, marital status, and income), financial distress, and 12-month MDE were measured. Logistic regression was used for data analysis. **Results:** In the pooled sample, 12-month MDE was associated with higher odds of financial distress, above and beyond objective SEP measures. We found MDE by race interaction on financial distress, suggesting stronger association between MDE and financial distress among African Americans, compared to Whites. **Conclusions:** The link between MDE and financial distress depends on race. The financial needs of African Americans with depression should be addressed. Depression screening is also needed for African Americans with financial distress.

## 1. Introduction

Theoretical work [1] and empirical data [2,3,4] have shown a close link between objective (e.g., wealth and income) and subjective (e.g., subjective social position) indicators of socioeconomic position (SEP) and health. The SEP-health association is bidirectional and involves causation and selection [1,5]. While SEP impacts mental health [1], psychiatric conditions such as depression impact SEP, via reducing individuals’ ability to work, earn income, and accumulate wealth [6]. Most past research, however, has focused on the effects of SEP indicators on depression, and less attention is paid to how depression increases financial distress. Using the National Survey of American Life (NSAL), this study compares African American and White adults for the association between experiencing a major depressive episode (MDE) and financial distress, one of the most widely accepted subjective measures of SEP [7].

Financial distress is a unique indicator of SEP. Although correlated with other SEP indicators [7], it is different enough from other conventional objective SEP indicators, such as education and income, in that it captures financial trouble that is a stronger antecedent to poor mental health than other objective SEP indicators that may not fully capture individuals’ material circumstances [8]. In support of this argument, financial distress has shown stronger associations with certain health outcomes than educational attainment, employment, or income [9,10,11]. Research has shown that financial distress conveys extra information over economic measures such as poverty status [12]. Some researchers have argued that financial distress reflects distinct economic characteristics that independently correlate with individuals’ and populations’ health beyond conventional SEP indicators [8,9,11,13,14,15,16]. Even among high-income individuals, financial distress correlates with health and health behaviors [8]. Trouble paying bills is associated with high risk behaviors [17]. The policy implication of research on financial distress is that standard SEP indicators should not be the only criteria for eligibility to the economic social programs and interventions [13,18,19,20]. Some populations may still experience financial distress despite high income and education, because they have lower disposable income. So, these families may have more difficulty paying bills and affording adequate food [21], which may have different effects than objective SEP indicators [9,19,22]. While African Americans are known to have higher levels of financial distress than Whites [23,24], we know very little about differences in the associations between financial distress and mental health of Whites and African Americans. 

Most theoretical work on this topic is focused on social causation. Link and Phelan’s Fundamental Cause Theory (FCT) conceptualized SEP as an upstream determinant of population health [1]. Mirowsky and Ross argued that the health effects of SEP are “enduring, consistent, and growing” [25]. While the health effects of objective measure of SEP (e.g., income, education, employment) are well documented [26,27,28,29,30], less is known about the association between subjective indicators of SEP, particularly financial distress, and health, after adjustment for SEP indicators. Specifically, less is known about subpopulation differences in the association between subjective SEP indicators and depression. 

Racial groups may differ in the effects of mental health problems, such as depression, on SEP indicators. Due to their worse access to the health care system, higher stigma, and a lower quality of treatment by the healthcare system [31], depression may be more disabling for African Americans than Whites [32]. In the NSAL data, Williams et al., showed that depression is more chronic for African Americans than Whites. They also found that African Americans have a higher tendency to rate their depression as severe and disabling compared to White Americans [32]. Other studies documented that compared to Whites with depression, African Americans with depression have higher levels of depressive symptoms and psychological distress [33,34]. All of this evidence suggests that depression is more chronic, severe, and disabling among African Americans than Whites.

Considerable racial variations may exist in the associations between mental health outcomes and SEP indicators [26,27,28,29,30]. Although overall, better health is associated with higher SEP in the overall population [5,29], this association may depend on the presence of other SEP indicators [27,35,36,37,38]. The link between SEP and health may vary based on SEP indicator, health outcome, and population [38,39,40,41,42]. Unfortunately, there is very limited knowledge on how Whites and African Americans differ in the magnitude of the association between depression and financial distress. 

Using NSAL data, a national sample of American adults, this study compared African Americans and Whites for the association between MDE and financial distress.

## 2. Methods

### 2.1. Design

The current study used data from the NSAL 2003-2004, which a component of the Collaborative Psychiatric Epidemiology Surveys (CPES) [43,44,45]. Although detailed description of the CPES and NSAL methodology is published elsewhere [43,44,45], here we provide some aspects of the NSAL methods.

### 2.2. Ethics

The NSAL/CPES received ethical approval from the University of Michigan (UM) Institute Review Board (IRB # B03-00004038-R1). NSAL participants signed a written informed consent. All procedures performed in the study were in accordance with the ethical standards of the institutional and/or national research committee and with the 1964 Helsinki Declaration and its later amendments or comparable ethical standards. 

### 2.3. Participants and Sampling

The NSAL sample was household probability sampling. African American and White adults were drawn from urban/rural areas and large cities. 

### 2.4. Interviews

From all interviews, 86% were face-to-face interviews. The remaining 14% of the interviews were telephone interviews. On average, interviews took about two hours and twenty minutes. All the interviews were performed in English language. The overall response rate of the NSAL study was 72%.

### 2.5. Measures

The current study collected data on race/ethnicity, age, gender, objective SEP (educational attainment, household income, employment status, and marital status), subjective SEP (i.e., financial distress), and 12-month MDE.

*Race/Ethnicity.* Self-identified race/ethnicity were measured. Individuals self-identified as African American when they were Black but did not report ancestral tie to the Caribbean countries. All the individuals with ancestries from Caribbean countries (Caribbean Black or Black Caribbean) were excluded from the current analysis.

*Financial Distress.* Financial distress was measured using the following two items: (1) “How difficult is it for (you/your family) to meet the monthly payments on your (family’s) bills?”, and (2) “How much do you worry that your total (family) income will not be enough to meet your (family’s) expenses and bills?” Response items for the first item ranged from 0 (not at all difficult) to 4 (extremely difficult). Responses for the second item ranged from 0 (not at all) to 4 (a great deal). Items were positively and strongly correlated (*r* = 0.645). We reverse coded the items, and calculated a total score, that ranged from 0 to 7, with a higher score indicating higher financial distress. A cut point of 0.80 (mean) was used to dichotomize financial distress.

*Major Depressive Episode (MDE).* 12-month MDE was measured using the modified version of the World Mental Health (WHO) Composite International Diagnostic Interview (CIDI). CIDI is a fully structured diagnostic interview schedule [46]. The CIDI measures a wide range of psychiatric disorders based on the Diagnostic and Statistical Manual, Fourth Edition (DSM-IV) criteria. Originally developed for the WHO study started in 2000, the CIDI is an interview schedule that is being used by lay interviewers who were trained. CIDI generates potential lifetime and recent diagnoses of non-psychotic psychiatric disorders [47]. Acceptable concordance between CIDI -diagnosis and blind clinical diagnosis by clinicians are shown in reappraisal studies [46,47,48,49], particularly for MDE [48]. CIDI provides valid diagnoses for African Americans and Whites [50,51].

*Confounders.* Socio-demographic factors included age (years), gender (male = 0, female = 1), educational attainment, household income, employment status, marital status. Household income was measured in (USD10,000) and treated as a continuous measure. Thus, a mean household income of 4.6 would indicate income of 46,000 USD. Employment status was a dichotomous variable (employed versus unemployed/not in labor market). Education attainment was treated as a dichotomous variable, with the following coding: 11 Years or Less = 0, 12 Years or More = 1. Marital status was operationalized as a dichotomous variable (0 = other status, 1 = married).

### 2.6. Statistical Analysis

Stata 13.0 (Stata Corp., College Station, TX) was used for analysis of the data. Taylor series approximation was used to re-estimate the complex design-based variance. All means, proportions, and standard errors reflect the weights due to the sampling complex design. As a result, the results are representative to the nation. Multivariable logistic regressions were applied for multivariable analysis. In the main model, 12-month MDE was the predictor, financial distress was the outcome, and confounders were controlled. We ran models in the absence and presence of interaction terms between race/ethnicity and MDE. Adjusted odds ratios (OR), 95% Confidence Intervals (CI), and their associated *p* values were reported. All *p* values equal or less than 0.05 were considered as statistically significant.

### 2.7. Sensitivity Analysis

To run a sensitivity analysis, we also ran models with financial distress as the predictor and 12-month MDE as the outcome. The results of this secondary analysis are available in the Appendix A.

## 3. Results

### 3.1. Descriptive Statistics

Table 1 describes age, objective SEP indicators (education, household income, marital status, and employment), financial distress, and 12-month MDE overall and based on race/ethnicity. Household income and education were lower among African Americans than Whites. Financial distress was higher among African Americans compared to Whites. (Table 1)

### 3.2. Logistic Regressions

*Financial distress as the Outcome.*Table 2 summarizes the result of a logistic regression with 12-month MDE as the independent and financial distress as the dependent variable. In the pooled sample 12-month MDE was linked with the odds of financial distress (OR = 2.11, 95%CI = 1.54–2.89), above and beyond all confounders. We found an interaction between race/ethnicity and 12-month MDE (OR = 1.84, 95%CI = 1.03–3.30) on financial distress, suggesting a stronger association between financial distress and MDE for African Americans compared to Whites.

*12-Month MDE as the Outcome. *Table A1 provides the summary of a logistic regression with financial distress as the independent and 12-month MDE as the dependent variable. In the pooled sample, financial distress was associated with higher odds of 12-month MDE (OR = 2.12, 95%CI = 1.52–2.94), above and beyond confounders. We found an interaction between race/ethnicity and financial distress (OR = 1.85, 95%CI = 1.01–3.40) on 12-month MDE, suggesting a stronger association for African Americans compared to Whites (Table A1).

## 4. Discussion

We found racial variation in the link between 12-month MDE and financial distress among American adults. Last Year Depression was more strongly associated with financial distress in African Americans, compared to Whites. This racial difference could be replicated regardless of whether MDE or financial distress was conceptualized as the independent or dependent variables.

Our finding on the association between financial distress and MDE is in line with the Fundamental Cause Theory (FCT) [1,35,36], which emphasizes low SEP as an upstream social determinant of a wide range of health problems [35,36,52]. The health effects of SEP indicators are robust; however, these effects may be specific to populations and health outcomes [37]. Poor SEP increases behavioral risk factors and reduces access to resources that can buffer the effects of stress. High SEP helps people escape risk factors and minimize consequences when they are faced. At the same time, severe decline in health lowers SEP, as illness may interfere with maintaining employment, which is the main source of income [53].

Compared to Whites, African Americans have higher odds of experiencing financial distress in the presence of depression. A potential explanation for this finding is the pervasive racial gap in wealth and financial assets. Overall, African Americans with the same income and education as Whites usually have much less overall wealth (e.g., equity on a home, savings) on their own and in their extended families than do Whites [54,55,56,57,58,59,60,61,62,63]. As wealth and similar resources may buffer financial distress [64], we found stronger association between depression and financial distress in African Americans than Whites. Deep discussions on the racial gap in wealth and their implications for the health and well-being of African Americans are published by Darity, Hamilton Shapiro, Oliver, and others [54,55,56,57,58,59,60,61,62,63]. Such inequalities are consequential for Whites as well [65].

Upward social mobility tends to be more taxing for African Americans compared to Whites [66,67]. As SEP is positively associated with discrimination for African Americans [68,69,70,71], the health gains that follow upward social mobility are smaller for African Americans [67,72]. In a recent study, parental education improved mental health of White but not African American young adults [60]. In another study, parental education had a stronger effect on educational mobility of Whites than African Americans [61]. While social mobility alters exposure to stress for Whites, African Americans report high levels of stress regardless of their social mobility status [62]. Other studies have also shown that upward social mobility may be differently linked to physical and mental health of Whites and African Americans [57,58,59,60,61,62,63,64,65,68,73,74,75] That is, upwardly mobile Whites report better health status than upwardly mobile African Americans [57,58,59]. These processes may help us understand why SEP and depression are differently linked by race.

In line with this argument, recent research has revealed high risk of depression among African Americans with high SEP [37,76,77]. In a study, income protected Whites but not African Americans against depression [54]. In the Fragile Families data, high family income reduced Attention Deficit Hyperactivity Disorder (ADHD) of White but not African American youth [77]. In NSAL data, high income was a risk factor for clinical depression among African American men [38]. In NSAL-Adolescents, high family income was a risk factor for MDE among African American males [6]. In the Americans Changing Lives study, higher education degree functioned as a risk factor for an increase in subsequent depression in African American men [76].

Several inconsistencies exist in the literature regarding the link between SEP and depression [72,76,77], reflecting the complex and non-linear nature of such association. One example is the positive link between family income and MDE risk in male African American youth [78] and adults [79]. High education credentials predicted an increase in depressive symptomatology among African American men, a finding which was missing for African American women or White men, or White women [76]. Similar inconsistencies are also reported for the effects of education and income on insomnia, physical inactivity, obesity [37,80,81], drinking [82], chronic disease [83] and mortality [84].

Our finding that MDE is associated with more financial distress is in contrast to previous research which has shown a systemic resilience toward socioeconomic adversities and stress in African Americans [85,86,87]. In a study, stressful life events showed a stronger association with MDE in Whites than in African Americans [85]. Physical health effects of economic adversities also seem to be larger for Whites than African Americans [88]. Review articles have shown that Whites may be more susceptible to several social and economic risk factors [86,87]. Thus, while African Americans seem to be more resilient to stress than Whites overall, this is not true for the effect of MDE on financial distress.

Our finding is also in support of the observations that depression is more disabling and chronic for African Americans than Whites [32,33,89]. Cross-sectional studies have shown that MDE is associated with more severe depressive symptoms in African Americans than Whites [32,33,89]. These pieces of evidence collectively suggest that African Americas with MDE have more financial distress than their White counterparts.

High financial distress in African Americans with MDE may be due to negative attitudes toward help-seeking and also differential treatment by the healthcare system. These differences may result in lower quality of depression treatment for African Americans than Whites [89]. African Americans have higher levels of stigma and preference toward non-pharmacological approaches to depression treatment [90,91]. In addition, given that comorbidities are more common in MDE among African Americans than Whites [91,92], MDE may be linked to more disability for African Americans. In addition, African Americans with MDE are more likely to receive depression treatment from their primary care physicians, which means a lower quality of treatment [93]. All these differences may result in more disability for African Americans with MDE than Whites with MDE [32]. In addition, a large proportion of African Americans report high levels of discrimination by the healthcare system, which predicts poor outcomes [50].

In this study, when additive (combined) effects of race and objective SEP on subjective SEP (financial distress) were tested, low education, unemployment, and low income but not race were associated with high level of financial distress. This finding suggests that the only reasons Blacks experience lower subjective SEP (i.e., financial distress) is their lower objective SEP. This finding may have policy implication that policies that enhance objective SEP may help African Americans perceive higher levels of fairness, as they find their social status as higher and the society as more just/fair [64,74,75].

When we had financial distress as the outcome, in addition to depression, objective SEP indicators (education, employment, and income) were correlated with financial distress. This suggests that (education, employment, and income impact financial distress, which is in line with the literature of the link between subjective and objective SEP indicators. However, when in our model, MDE was the outcome, in the presence of financial distress, objective SEP indicators (education, income, marital status, and unemployment) did not have residual effects on MDE. This finding of our sensitivity analysis suggests that financial distress, an indicator of subjective SEP, may have stronger association with MDE than objective indicators of SEP.

### Limitations

The current study had a few methodological and conceptual limitations. First, due to the cross-sectional design, the current study cannot draw any causative inference, thus future research should test whether African Americans may have higher vulnerability to the effects of financial distress on MDE, or if their MDE results in larger financial scar. Second, financial distress was measured using only two items. Future research may use comprehensive measures to investigate financial distress. Third, this study did not include participation in governmental or state welfare programs, such as Temporary Assistance for Needy Families (TANF) and Supplemental Nutrition Assistance Program (SNAP) [94,95,96,97,98]. Finally, other sources of stress, such as food insecurity, hunger, and stressful life events, were not measured. Despite these limitations, the study still offers new knowledge to the scientific community with the following strengths: (1) a large sample size; (2) national sampling; and (3) high validity of depression measure.

## 5. Conclusions

To conclude, the association between 12-month MDE and financial distress among American adults depends on race. That is, compared to Whites, African Americans have higher odds of experiencing financial distress in the presence of depression. As a result, financial distress may be a more important aspect of screening, diagnosis, and treatment of depression for African Americans than Whites.

## Figures and Tables

**Table 1:** Descriptive statistics in the pooled sample.

Characteristics	All		Whites		African Americans	
	*Mean*	*95% CI*	*Mean*	*95% CI*	*Mean*	*95% CI*
Age (Years)	43.57	42.11–45.02	44.84	41.96–47.73	42.20	41.14–43.26
Household Income (USD10,000)	4.17	3.75–4.58	4.68	3.89–5.46	3.62	3.35–3.89
Financial Distress	1.82	1.71–1.93	1.71	1.52–1.90	1.94	1.82–2.05
	**%**	**95% CI**	**%**	**95% CI**	**%**	**95% CI**
Gender						
Male	45.76	43.59–47.94	47.38	43.27–51.53	44.00	42.32–45.70
Female	54.24	52.06–56.41	52.62	48.47–56.73	56.00	54.30–57.68
Education						
11 Years or Less	19.47	16.75–22.51	15.16	10.68–21.08	24.11	21.79–26.61
12 Years or More	80.53	77.49–83.25	84.84	78.92–89.32	75.89	73.39–78.21
Unemployed						
No	92.73	91.06–94.12	95.48	92.28–97.39	89.77	88.18–91.17
Yes	7.27	5.88–8.94	4.52	2.61–7.72	10.23	8.83–11.82
Married						
No	51.78	47.94–55.61	45.81	38.62–53.17	58.23	56.08–60.36
Yes	48.22	44.39–52.06	54.19	46.83–61.38	41.77	39.64–43.92
12-Month MDE						
No	92.68	91.76–93.52	92.12	90.46–93.51	93.30	92.29–94.18
Yes	7.32	6.48–8.24	7.88	6.49–9.54	6.70	5.82–7.71

MDE; Major Depressive Episode.

**Table 2:** Summary of logistic regressions with major depressive episode (MDE) as the independent variable and financial distress as the dependent variable.

	Model 1		Model 2	
	OR	95% CI	OR	95% CI
Race (African American)	1.08	0.82–1.41	1.04	0.79–1.36
Gender (Female)	1.38 ***	1.14–1.67	1.37 **	1.13–1.66
Age	0.98 ***	0.97–0.98	0.98 ***	0.97–0.98
Education (>=12 Years)	0.59 ***	0.43–0.80	0.59 ***	0.43–0.81
Unemployed	1.83 **	1.20–2.80	1.83 **	1.19–2.80
Married	1.20	0.87–1.66	1.21	0.88–1.66
Income (USD10,000)	0.86 ***	0.82–0.91	0.86 ***	0.82–0.91
12-Month MDE	2.11 ***	1.54–2.89	1.66 *	1.09–2.55
12-Month MDE × Race	-	-	1.84 *	1.03–3.30
Intercept	4.94 ***	3.15–7.74	5.03 ***	3.20–7.93

Outcome: Financial Distress, OR; Odds Ratio, CI; Confidence Interval, MDE; Major Depressive Episode, * *p* < 0.05; ** *p* < 0.01; *** *p* < 0.001.

## Appendix A

**Table A1:** Summary of logistic regressions with financial distress as the independent variable and MDE as the dependent variable.

	Model 1		Model 2	
	OR	95% CI	OR	95% CI
Race (African Americans)	0.72 **	0.57–0.90	0.47 **	0.29–0.75
Gender (Female)	1.01	0.64–1.59	1.01	0.64–1.60
Age	0.99	0.97–1.01	0.99	0.97–1.01
Education (>=12 Years)	0.89	0.55–1.42	0.89	0.55–1.43
Unemployed	1.01	0.63–1.64	1.00	0.62–1.62
Married	0.79	0.45–1.37	0.79	0.46–1.36
Income (USD10000)	0.96	0.90–1.03	0.96	0.90–1.03
Financial Distress	2.12 ***	1.52–2.94	1.65 *	1.02–2.67
Financial Distress × Race	-	-	1.85 *	1.01–3.40
Intercept	0.12 ***	0.05–0.29	0.15 ***	0.07–0.34

Outcome: MDE; Major Depressive Episode. * *p* < 0.05; ** *p* < 0.01; *** *p* < 0.001.

## References

[1] Phelan J.C., Link B.G., Tehranifar P. (2010). Social conditions as fundamental causes of health inequalities: Theory, evidence, and policy implications. J. Health Soc. Behav..

[2] Conti G., Heckman J., Urzua S. (2010). The education-health gradient. Am. Econ. Rev..

[3] Baker D.P., Leon J., Smith Greenaway E.G., Collins J., Movit M. (2011). The education effect on population health: A reassessment. Popul. Dev. Rev..

[4] Davey S., Hart C., Hole D., MacKinnon P., Gillis C., Watt G., Blane D., Hawthorne V. (1998). Education and occupational social class: Which is the more important indicator of mortality risk?. J. Epidemiol. Community Health.

[5] Warren J.R. (2009). Socioeconomic Status and Health across the Life Course: A Test of the Social Causation and Health Selection Hypotheses. Soc. Forces.

[6] Mulatu M.S., Schooler C. (2002). Causal connections between socio-economic status and health: Reciprocal effects and mediating mechanisms. J. Health Soc. Behav..

[7] Conklin A.I., Forouhi N.G., Suhrcke M., Surtees P., Wareham N.J., Monsivais P. (2013). Socioeconomic status, financial hardship and measured obesity in older adults: A cross-sectional study of the EPIC-Norfolk cohort. BMC Public Health.

[8] Rahkonen O., Laaksonen M., Karvonen S. (2005). The contribution of lone parenthood and economic difficulties to smoking. Soc. Sci. Med..

[9] Laaksonen M., Sarlio-Lähteenkorva S., Lahelma E. (2004). Multiple dimensions of socioeconomic position and obesity among employees: The Helsinki Health Study. Obes. Res..

[10] Starkey A.J., Keane C.R., Terry M.A., Marx J.H., Ricci E.M. (2013). Financial distress and depressive symptoms among African American women: identifying financial priorities and needs and why it matters for mental health. J. Urban Health..

[11] Loman T., Lallukka T., Laaksonen M., Rahkonen O., Lahelma E. (2013). Multiple socioeconomic determinants of weight gain: The Helsinki Health Study. BMC Public Health.

[12] Ouellette T., Burstein N., Long D., Beecroft E. (2004). Measures of Material Hardship: Final Report.

[13] Laaksonen E., Lallukka T., Lahelma E., Ferrie J.E., Rahkonen O., Head J., Marmot M.G., Martikainen P. (2011). Economic difficulties and physical functioning in Finnish and British employees: Contribution of social and behavioural factors. Eur. J. Public Health.

[14] Laaksonen E., Martikainen P., Lallukka T., Lahelma E., Ferrie J., Rahkonen O., Marmot M., Head J. (2009). Economic difficulties and common mental disorders among Finnish and British white-collar employees: The contribution of social and behavioural factors. J. Epidemiol. Commun. Health.

[15] Ferrie J., Martikainen P., Shipley M., Marmot M. (2005). Self-reported economic difficulties and coronary events in men: Evidence from the Whitehall II study. Int. J. Epidemiol..

[16] Lallukka T., Ferrie J.E., Rahkonen O., Shipley M.J., Pietiläinen O., Kivimäki M., Marmot M.G., Lahelma E. (2013). Change in economic difficulties and physical and mental functioning: Evidence from British and Finnish employee cohorts. Scand. J. Work Environ. Health.

[17] Averett S.L., Smith J.K. (2013). Financial hardship and obesity: The link between weight and household debt. Women.

[18] Braveman P., Cubbin C., Egerter S., Chideya S., Marchi K., Metzler M., Posner S. (2005). Socioeconomic status in health research: One size does not fit all. JAMA.

[19] Kahn J.R., Pearlin L.I. (2006). Financial strain over the life course and health among older adults. J. Health Soc. Behav..

[20] Lallukka T., Ferrie J.E., Kivimäki M., Shipley M.J., Rahkonen O., Lahelma E. (2012). Economic difficulties and subsequent sleep problems: Evidence from British and Finnish occupational cohorts. Sleep Med..

[21] Office of National Statistics (2011). Family Spending 2011 Edition: A Report on the Living Costs and Food Survey 2010.

[22] Pudrovska T., Schieman S., Pearlin L.I., Nguyen K. (2005). The sense of mastery as a mediator and moderator in the association between economic hardship and health in late life. J. Aging Health.

[23] Lincoln K.D., Chatters L.M., Taylor R.J. (2003). Psychological distress among black and white Americans: Differential effects of social support, negative interaction and personal control. J. Health Soc. Behav..

[24] Hall M.H., Matthews K.A., Kravitz H.M., Gold E.B., Buysse D.J., Bromberger J.T., Owens J.F., Sowers M. (2009). Race and financial strain are independent correlates of sleep in midlife women: The SWAN sleep study. Sleep.

[25] Mirowsky J., Ross C.E. (2003). Education, Social Status, and Health.

[26] Bowen M.E., González H.M. (2010). Childhood socioeconomic position and disability in later life: Results of the health and retirement study. Am. J. Public Health.

[27] Herd P., Goesling B., House J.S. (2007). Socioeconomic position and health: The differential effects of education versus income on the onset versus progression of health problems. J. Health Soc. Behav..

[28] Kim J. (2008). Intercohort trends in the relationship between education and health: Examining physical impairment and depressive symptomatology. J. Aging Health.

[29] Leopold L., Engelhardt H. (2013). Education and physical health trajectories in old age. Evidence from the Survey of Health, Ageing and Retirement in Europe (SHARE). Int. J. Public Health.

[30] Johnson-Lawrence V.D., Griffith D.M., Watkins D.C. (2013). The effects of race, ethnicity and mood/anxiety disorders on the chronic physical health conditions of men from a national sample. Am. J. Mens Health.

[31] Watkins D.C., Abelson J.M., Jefferson S.O. (2013). “Their depression is something different… it would have to be”: Findings from a qualitative study of black women’s perceptions of depression in black men. Am. J. Mens Health.

[32] Williams D.R., González H.M., Neighbors H., Nesse R., Abelson J.M., Sweetman J., Jackson J.S. (2007). Prevalence and distribution of major depressive disorder in African Americans, Caribbean blacks, and non-Hispanic whites: Results from the National Survey of American Life. Arch. Gen. Psychiatry.

[33] Assari S., Moazen-Zadeh E. (2016). Ethnic Variation in the Cross-sectional Association between Domains of Depressive Symptoms and Clinical Depression. Front. Psychiatry.

[34] Watkins D.C., Johnson N.C. (2018). Age and Gender Differences in Psychological Distress among African Americans and Whites: Findings from the 2016 National Health Interview Survey. Healthcare.

[35] Link B.G., Phelan J., Bird C.E., Conrad P., Fremont A.M., Timmermans S. (2010). Social conditions as fundamental causes of health inequalities. Handbook of Medical Sociology.

[36] Link B., Phelan J. (1995). Social conditions as fundamental causes of disease. J. Health Soc. Behav..

[37] Assari S., Nikahd A., Malekahmadi M.R., Lankarani M.M., Zamanian H. (2016). Race by gender group differences in the protective effects of socioeconomic factors against sustained health problems across five domains. J. Racial Ethn. Health Disparities.

[38] Hudson D.L., Neighbors H.W., Geronimus A.T., Jackson J.S. (2012). The relationship between socioeconomic position and depression among a US nationally representative sample of African Americans. Soc. Psychiatry Psychiatr. Epidemiol..

[39] Martikainen P., Lahelma E., Ripatti S., Albanes D., Virtam J. (2001). Educational differences in lung cancer mortality in male smokers. Int. J. Epidemiol..

[40] Steenland K., Henley J., Thun M. (2002). All-cause and cause-specific death rates by educational status for two million people in two American Cancer Society cohorts, 1959–1996. Am. J. Epidemiol..

[41] Boardman J.D., Fletcher J.M. (2015). To cause or not to cause? That is the question, but identical twins might not have all of the answers. Soc. Sci. Med..

[42] Mackenbach J.P., Stirbu I., Roskam A.J., Schaap M.M., Menvielle G., Leinsalu M., Kunst A.E. (2008). European Union Working Group on Socioeconomic Inequalities in Health. Socioeconomic inequalities in health in 22 European countries. N. Engl. J. Med..

[43] Jackson J.S., Neighbors H.W., Nesse R.M., Trierweiler S.J., Torres M. (2004). Methodological innovations in the National Survey of American Life. Int. J. Methods Psychiatr. Res..

[44] Jackson J.S., Torres M., Caldwell C.H., Neighbors H.W., Nesse R.M., Taylor R.J., Trierweiler S.J., Williams D.R. (2004). The National Survey of American Life: A study of racial, ethnic and cultural influences on mental disorders and mental health. Int. J. Methods Psychiatr. Res..

[45] Heeringa S.G., Wagner J., Torres M., Duan N., Adams T., Berglund P. (2004). Sample designs and sampling methods for the Collaborative Psychiatric Epidemiology Studies (CPES). Int. J. Methods Psychiatr. Res..

[46] Hu W. (1994). Reliability and validity studies of the WHO-Composite International Diagnostic Interview (CIDI): A critical review. J. Psychiatr. Res..

[47] Robins L.N., Wing J., Wittchen H.U., Helzer J.E., Babor T.F., Burke J., Farmer A., Jablenski A., Pickens R., Regier D.A. (1988). The Composite International Diagnostic Interview. An epidemiologic instrument suitable for use in conjunction with different diagnostic systems and in different cultures. Arch. Gen. Psychiatry.

[48] Kessler R.C., Calabrese J.R., Farley P.A., Gruber M.J., Jewell M.A., Katon W., Keck P.E., Nierenberg A.A., Sampson N.A., Shear M.K. (2013). Composite International Diagnostic Interview screening scales for DSM-IV anxiety and mood disorders. Psychol. Med..

[49] Kessler R.C., Wittchen H.-U., Abelson J.M., McGonagle K., Schwarz N., Kendler K.S., Knäuper B., Zhao S. (1998). Methodological studies of the Composite International Diagnostic Interview (CIDI) in the US National Comorbidity Survey. Int. J. Methods Psychiatr. Res..

[50] Williams D.R., Haile R., González H.M., Neighbors H., Baser R., Jackson J.S. (2007). The mental health of Black Caribbean immigrants: Results from the National Survey of American Life. Am. J. Public Health.

[51] Neighbors H.W., Caldwell C., Williams D.R., Nesse R., Taylor R.J., Bullard K.M., Torres M., Jackson J.S. (2007). Race, ethnicity, and the use of services for mental disorders: Results from the National Survey of American Life. Arch. Gen. Psychiatry.

[52] Freese J., Lutfey K., Pescosolido B.A., Martin J.K., McLeod J.D., Rogers A. (2010). Fundamental causality: Challenges of an animating concept for medical sociology. Handbook of the Sociology of Health, Illness, and Healing: A Blueprint for the 21st Century.

[53] Singh-Manoux A., Clarke P., Marmot M. (2002). Multiple measures of socio-economic position and psychosocial health: proximal and distal measures. Int. J. Epidemiol..

[54] Muñoz A.P., Kim M., Chang M., Jackson R., Hamilton D., Darity W.A. The Color of Wealth in Boston.

[55] Hamilton D., Darity W.A. (2017). The Political Economy of Education, Financial Literacy, and the Racial Wealth Gap; Economic Research. Review.

[56] Hamilton D., Darity W. (2009). Race, Wealth, and Intergenerational Poverty: There will never be a post-racial America if the wealth gap persists. Am. Prospect.

[57] Hamilton D., Darity W., Price A.E., Sridharan V., Tippett R. (2015). Umbrellas Don’t Make it Rain: Why Studying and Working Hard Isn’t Enough for Black Americans.

[58] De La Cruz-Viesca M., Chen Z., Ong P.M., Hamilton D., Darity W.A. (2016). The Color of Wealth in Los Angeles.

[59] Oliver M.L., Shapiro T.M. (1995). A Sociology of Wealth and Racial Inequality. Redress for Historical Injustices in the United States: On Reparations for Slavery, Jim Crow, and Their Legacies.

[60] Oliver M.L., Shapiro T.M. (1990). Wealth of a Nation: A Reassessment of Asset Inequality in America Shows At Least One Third of Households Are Asset-poor. Am. J. Econ. Sociol..

[61] Oliver M.L., Shapiro T.M. (2001). Wealth and racial stratification. Am. Becom..

[62] Shapiro T., Meschede T., Osoro S. (2013). The Roots of the Widening Racial Wealth Gap: Explaining the Black-White Economic Divide.

[63] Oliver M., Shapiro T. (2013). Black Wealth/White Wealth: A New Perspective on Racial Inequality.

[64] Overby L.M., Brown R.D., Bruce J.M., Smith C.E., Winkle J.W. (2004). Justice in black and white: Race, perceptions of fairness, and diffuse support for the judicial system in a southern state. Justice Syst. J..

[65] Malat J., Mayorga-Gallo S., Williams D.R. (2018). The effects of whiteness on the health of whites in the USA. Soc. Sci. Med..

[66] Fuller-Rowell T.E., Doan S.N. (2010). The social costs of academic success across ethnic groups. Child Dev..

[67] Fuller-Rowell T.E., Curtis D.S., Doan S.N., Coe C.L. (2015). Racial disparities in the health benefits of educational attainment: A study of inflammatory trajectories among African American and white adults. Psychosom. Med..

[68] Assari S., Lankarani M.M., Caldwell C.H. (2018). Does Discrimination Explain High Risk of Depression among High-Income African American Men?. Behav. Sci..

[69] Assari S., Moghani Lankarani M. (2018). Workplace Racial Composition Explains High Perceived Discrimination of High Socioeconomic Status African American Men. Brain Sci..

[70] Assari S. (2018). Does School Racial Composition Explain Why High Income Black Youth Perceive More Discrimination? A. Gender Analysis. Brain Sci..

[71] Assari S., Gibbons F.X., Simons R.L. (2018). Perceived Discrimination among Black Youth: An 18-Year Longitudinal Study. Behav. Sci..

[72] Hudson D.L., Bullard K.M., Neighbors H.W., Geronimus A.T., Yang J., Jackson J.S. (2012). Are benefits conferred with greater socioeconomic position undermined by racial discrimination among African American men?. J. Mens. Health.

[73] Smith D.M., Langa K.M., Kabeto M.U., Ubel P.A. (2005). Health, wealth, and happiness: Financial resources buffer subjective well-being after the onset of a disability. Psychol. Sci..

[74] Sigelman L., Welch S. (1994). Black Americans’ Views of Racial Inequality: The Dream Deferred.

[75] Weatherspoon F.D. (2006). Racial justice and equity for African-American males in the American educational system: A dream forever deferred. NC Cent. Law J..

[76] Assari S. (2017). Combined racial and gender differences in the long-term predictive role of education on depressive symptoms and chronic medical conditions. J. Racial Ethn. Health Disparities.

[77] Assari S., Caldwell C.H. (2019). Family Income at Birth and Risk of Attention Deficit Hyperactivity Disorder at Age 15: Racial Differences. Children.

[78] Assari S., Caldwell C.H. (2017). High Risk of Depression in High-Income African American Boys. J. Racial Ethn. Health Disparities.

[79] Assari S. (2017). Social Determinants of Depression: The Intersections of Race, Gender, and Socioeconomic Status. Brain Sci..

[80] Assari S., Thomas A., Caldwell C.H., Mincy R.B. (2017). Blacks’ Diminished Health Return of Family Structure and Socioeconomic Status; 15 Years of Follow-up of a National Urban Sample of Youth. J. Urban Health.

[81] Assari S. (2016). Psychosocial Correlates of Body Mass Index in the United States: Intersection of Race, Gender and Age. Iran. J. Psychiatry Behav. Sci..

[82] Assari S., Lankarani M.M. (2016). Education and Alcohol Consumption among Older Americans; Black-White Differences. Front. Public Health.

[83] Assari S. (2018). The Benefits of Higher Income in Protecting against Chronic Medical Conditions Are Smaller for African Americans than Whites. Healthcare.

[84] Assari S., Lankarani M.M. (2016). Race and Urbanity Alter the Protective Effect of Education but not Income on Mortality. Front. Public Health.

[85] Assari S., Lankarani M.M. (2016). Association between Stressful Life Events and Depression; Intersection of Race and Gender. J. Racial Ethn. Health Disparities.

[86] Assari S. (2018). Health Disparities Due to Blacks’ Diminished Return: Public Policy Solutions. Soc. Issues Policy Rev..

[87] Assari S. (2017). Unequal gain of equal resources across racial groups. Int. J. Health Policy Manag..

[88] Geronimus A.T., Pearson J.A., Linnenbringer E., Schulz A.J., Reyes A.G., Epel E.S., Lin J., Blackburn E.H. (2015). Race-Ethnicity, Poverty, Urban Stressors, and Telomere Length in a Detroit Community-based Sample. J. Health Soc. Behav..

[89] Cooper L.A., Gonzales J.J., Gallo J.J., Rost K.M., Meredith L.S., Rubenstein L.V., Wang N.Y., Ford D.E. (2003). The acceptability of treatment for depression among African-American, Hispanic, and white primary care patients. Med. Care.

[90] Brown C., Conner K.O., Copeland V.C., Grote N., Beach S., Battista D., Reynolds C.F. (2010). Depression stigma, race, and treatment seeking behavior and attitudes. J. Community Psychol..

[91] Schmidt L., Greenfield T., Mulia N. (2006). Unequal treatment: Racial and ethnic disparities in alcoholism treatment services. Alcohol. Res. Health.

[92] Watkins D.C., Assari S., Johnson-Lawrence V. (2015). Race and ethnic group differences in comorbid major depressive disorder, generalized anxiety disorder, and chronic medical conditions. J. Racial Ethn. Health Disparities.

[93] Stockdale S.E., Lagomasino I.T., Siddique J., McGuire T., Miranda J. (2008). Racial and ethnic disparities in detection and treatment of depression and anxiety among psychiatric and primary health care visits, 1995–2005. Med. Care..

[94] Pavetti L., Derr M.K., Kauff J.F., Barrett A. (2010). Mental disorders and service use among welfare and disability program participants in fee-for-service. Medicaid. Psychiatr Serv..

[95] Coiro M.J. (2001). Depressive symptoms among women receiving welfare. Women Health.

[96] Petterson S.M., Friel L.V. (2001). Psychological distress, hopelessness and welfare. Women Health.

[97] Davidson T.C., Singelmann J. (2010). Determinants of Depressive Symptoms Among Women on Public Assistance in Louisiana. J. Health Dispar. Res. Pract..

[98] Hastings J.F., Snowden L.R. (2018). Mental health treatment and work among African American and Caribbean Black welfare recipients. Cultur. Divers. Ethnic Minor. Psychol..

